# Unraveling the Hexaploid Sweetpotato Inheritance Using Ultra-Dense Multilocus Mapping

**DOI:** 10.1534/g3.119.400620

**Published:** 2019-11-15

**Authors:** Marcelo Mollinari, Bode A. Olukolu, Guilherme da S. Pereira, Awais Khan, Dorcus Gemenet, G. Craig Yencho, Zhao-Bang Zeng

**Affiliations:** * Bioinformatics Research Center, North Carolina State University, Raleigh, North Carolina,; † Department of Horticultural Science, North Carolina State University, Raleigh, North Carolina,; ‡ Department of Entomology and Plant Pathology, University of Tennessee, Knoxville, Tennessee,; § Plant Pathology and Plant-Microbe Biology Section, Cornell University, Geneva, New York, and; ** International Potato Center, ILRI Campus, Nairobi, Kenya

**Keywords:** Polyploidy, Genetic Linkage, Hexasomic Inheritance, Haplotyping, Preferential Pairing, Multivalent

## Abstract

The hexaploid sweetpotato (*Ipomoea batatas* (L.) Lam., 2n = 6x = 90) is an important staple food crop worldwide and plays a vital role in alleviating famine in developing countries. Due to its high ploidy level, genetic studies in sweetpotato lag behind major diploid crops significantly. We built an ultra-dense multilocus integrated genetic map and characterized the inheritance system in a sweetpotato full-sib family using our newly developed software, MAPpoly. The resulting genetic map revealed 96.5% collinearity between *I. batatas* and its diploid relative *I. trifida*. We computed the genotypic probabilities across the whole genome for all individuals in the mapping population and inferred their complete hexaploid haplotypes. We provide evidence that most of the meiotic configurations (73.3%) were resolved in bivalents, although a small portion of multivalent signatures (15.7%), among other inconclusive configurations (11.0%), were also observed. Except for low levels of preferential pairing in linkage group 2, we observed a hexasomic inheritance mechanism in all linkage groups. We propose that the hexasomic-bivalent inheritance promotes stability to the allelic transmission in sweetpotato.

The cultivated hexaploid sweetpotato (*Ipomoea batatas* (L.) Lam., 2n = 6x = 90) is an important staple food crop worldwide with an annual production of ∼113 million tons (FAO 2017). It plays a vital role in alleviating famine, especially in developing countries in Africa and Southeast Asia (Loebenstein 2009). Despite its undeniable social and economic importance, genetic studies in sweetpotato significantly lag behind major diploid crops due to its complex polyploid genome. Polyploids are organisms with more than two complete sets of homologous chromosomes. They are grouped into two categories, allopolyploids or autopolyploids, when these chromosomes are originated from either different or from the same species, respectively (Comai 2005). While in diploid organisms the study of allelic transmission and genetic linkage are relatively simple, these studies are considerably complicated in polyploids due to the wide range of meiotic configurations these species undergo (Sybenga 1975; Gallais 2003; Zielinski and Scheid 2012). Moreover, current linkage analysis methods for complex polyploids (*i.e.*, ploidy level >4) are mostly based on pairwise (or two-point) marker analyses (Fisher 1941; Ripol *et al.* 1999; Kriegner *et al.* 2003; Aitken *et al.* 2007; Cervantes-Flores *et al.* 2008; van Geest *et al.* 2017). These methods rely on the assumption that the information in isolated pairs of markers is sufficient to detect recombination events between them accurately. In complex polyploids, however, this is rarely the case due to the limited mapping population size and the incomplete information provided by biallelic markers. Here, we present a fully informative multilocus genetic map of a full-sib hexaploid sweetpotato population derived from a cross between the cultivars ‘Beauregard’ and ‘Tanzania’ (BT population) scored with more than 30,000 informative single nucleotide polymorphisms (SNPs) using our newly developed R package called MAPpoly. We also inferred the haplotypes of all individuals in the full-sib population, which provided novel insights into the multivalent formation and preferential pairing in the sweetpotato genome.

Our multilocus analysis considers multiple SNPs simultaneously and propagates their information through the linkage group (LG) to overcome the typical low informativeness of some two-loci combinations. This strategy is fundamentally important for complex polyploid genome analysis since pairs of biallelic markers carry very little information about the recombination process individually (Luo *et al.* 2004; Mollinari and Garcia 2019). Moreover, the signal-to-noise (S/N) ratio in complex polyploid SNP data sets is considerably lower as compared to that in diploids and tetraploids (Mollinari and Serang 2015), thus making the genotype calling more prone to errors. The multilocus approach takes into account these errors by using the probability distribution of genotypes provided by the genotype calling software (Mollinari and Garcia 2019). Therefore, multilocus methods are essential to make appropriate use of the information of multiple-dose markers and assess complex polyploid inheritance systems.

Several studies attempted to elucidate the polyploidy nature in sweetpotato (allo *vs.* autopolyploid), including cytological and molecular marker analyses (Gustafsson and Gadd 1965; Magoon *et al.* 1970; Shiotani and Kawase 1987; Austin 1988; Ukoskit and Thompson 1997; Kriegner *et al.* 2003; Cervantes-Flores *et al.* 2008; Zhao *et al.* 2013; Monden and Tahara 2017), and more recently sequence-based studies (Roullier *et al.* 2013; Yang *et al.* 2017; Muñoz-Rodríguez *et al.* 2018). Two polyploidization scenarios were proposed: the first suggests an allopolyploid origin involving the hybridization of two sweetpotato wild diploid relatives, *I. trifida* and *I. triloba* (Austin 1988); the second, well supported by the literature, suggests an autopolyploid origin with *I. trifida* having a dual role in the polyploidization process (Shiotani and Kawase 1987; Roullier *et al.* 2013; Yang *et al.* 2017; Muñoz-Rodríguez *et al.* 2018). Corroborating this scenario, the polysomic inheritance observed in several molecular marker studies rules out the strict allopolyploid sweetpotato origin (Kriegner *et al.* 2003; Cervantes-Flores *et al.* 2008; Zhao *et al.* 2013; Monden and Tahara 2017).

Nevertheless, none of these studies presented a comprehensive profile of chromosomal pairing for all homology groups across the whole genome nor the potential formation of multivalents at a population level. Solving these missing pieces of information is essential to unravel the precise mode of inheritance in sweetpotato, and consequently, allow an efficient application of molecular techniques in this complex polyploid breeding system. The BT population coupled with high-coverage sequence genotyping used in this study has two essential characteristics that enabled high-quality mapping: 1) high and uniform sequence read depth across the genome, which allows for high-quality genotype calling including multidose markers, and 2) sufficiently large sample size to allow the detection of recombination events in a hexaploid scenario. Additionally, we considered the uncertainty in the genotype calling by modeling the error during the map construction using a hidden Markov model (HMM) (Mollinari and Garcia 2019). Moreover, all methods can be readily used in tetraploid and octoploid full-sib populations.

## Materials and Methods

### Plant material

The mapping population consists of 315 full-sib individuals originated from a cross between the oranged-flesh cultivar ‘Beauregard’ (CIP440132 - male) and the African landrace ‘Tanzania’ (CIP440166 - female). These two cultivars were selected due to their agronomic importance and contrasting traits, such as beta-carotene and dry matter contents, drought tolerance, and resistance for viruses and nematodes (Cervantes-Flores *et al.* 2008; Gemenet *et al.* 2019), for further QTL studies.

### Optimized genotyping-by-sequencing protocol - GBSpoly

Next-Generation Sequencing (NGS) library preparation protocol was optimized for polyploids and highly heterozygous genomes to produce uniform coverage across samples and loci, GBSpoly (Wadl *et al.* 2018) (details in S1 Extended Material and Methods). The optimizations were based on re-engineered barcoded adapters that ensure accurate demultiplexing and base calls. The 6-9 bp variable length barcodes were designed to account for both nucleotide substitution and indel errors (based on edit/levenshtein distance), to minimize phasing errors and to maintain nucleotide diversity at every position along the reads. We also introduced buffer sequences upstream of the barcodes to ensure that the barcodes lie in high-quality base regions by avoiding the elevated error rates at the ends of the reads. The adapters were ligated to fragments generated by double digests, *Tse*I and *Cvi*AII, and then size selected to minimize PCR bias. By designing barcodes that did not reconstitute the restriction sites, ligated fragments were subjected to a secondary digest to eliminate chimeric fragments. Sequencing was performed on the Illumina HiSeq 2500. For more details, see S1 Extended Material and Methods.

### Genotype calling

We used the software SuperMASSA (Serang *et al.* 2012) to perform the genotype calling of parents and offspring of the full-sib population. For quality control purposes, we eliminated SNPs with read depth <20 and estimated ploidy levels different from six. We also filtered out SNPs with more than 25% of missing data and with segregation distortion ($P<5\times 10^{-4}$). Additionally, we removed four individuals with less than 100 reads on average for the selected SNPs (see S1 Extended Material and Methods). We obtained the physical positions of the selected markers in two diploid reference genomes of *I. trifida* and *I. triloba* (Wu *et al.* 2018) and classified them into shared between both genomes or private to a specific genome based on the full-sib population genotype calls.

### De novo map construction

#### Grouping and SNP ordering:

We computed recombination fractions for all marker pairs considering all possible linkage phase configurations. For each marker pair, we selected the recombination fraction associated with the most likely linkage phase and assembled a recombination fraction matrix for all marker pairs. Using Unweighted Pair Group Method with Arithmetic Mean (UPGMA) hierarchical clustering, we generated a dendrogram representing 15 LGs corresponding to the 15 sweetpotato homology groups. To order the SNPs within each LG, we converted the recombination fractions to genetic distances using Haldane’s map function and applied the unconstrained Multidimensional Scaling (MDS) algorithm with the squared LOD Scores to construct the stress criterion (Preedy and Hackett 2016).

#### Phasing and multilocus map estimation:

The parental linkage phase configuration was obtained by serially adding markers to the map sequence and evaluating two-point likelihoods associated with possible configurations between the inserted markers and the ones already positioned. If the LOD Score between the two most likely configurations was less than ten for a subset of configurations, we compared the multipoint likelihoods of these phase configurations to proceed to the next marker insertion. When the last marker was inserted, we re-estimated the multipoint recombination fractions between all adjacent markers (Mollinari and Garcia 2019). For more details, see S1 Extended Material and Methods.

### Reference genome-assisted map improvement

Using the *I. trifida* reference, we detected collinearity blocks within each LG by visually inspecting abrupt breakages in the scatter plots continuity (Supplemental Material, S5 Fig.). For each collinearity block, we evaluated the multilocus likelihood associated with the initial MDS-based “*de novo*” order and the order provided by *I. trifida* reference. We selected the maximum likelihood order for each block, tested several orientations among them (S1 File), and chose the configuration that yielded the highest multilocus likelihood for the complete map. Next, we inserted the remaining private SNPs from *I. triloba* using the genomic position constraints imposed by SNPs shared by both genomes. We also eliminated SNPs that caused substantial map expansions (see S1 Extended Material and Methods). Finally, we re-estimated the map by considering the probability distribution of the genotype calling provided by SuperMASSA (Serang *et al.* 2012). We also computed the Genotypic Information Content (GIC) (Bourke *et al.* 2018) for each homolog across the entire genome.

### Probability distribution of the offspring genotypes

The probability distribution for all possible 400 hexaploid genotypes was calculated using the HMM framework detailed in S1 Extended Material and Methods. Briefly, if $G_{k,j}$ denote the *j*^th^ genotype, j∈1,⋯,400, of an individual in a hexaploid full-sib population at locus *k*, the conditional probability distribution of $G_{k,j}$ is defined as

$$\text{Pr}\left(G_{k,j}|O,\lambda \right)=\frac{\alpha_{k}\left(j\right)\beta_{k}\left(j\right)}{\sum_{i=1}^{400}\alpha_{k}\left(i\right)\beta_{k}\left(i\right)}$$ (1)

where $O=\left\{O_{1},\cdots ,O_{z}\right\}$ is a sequence of observations of *z* markers, *λ* denotes the map parameters, $\alpha_{k}\left(j\right)$ denotes the joint probability of the partial observation sequence to the left of marker *k* (including *k*) and genotype $G_{k,j}$, given the map parameters *λ*; similarly, $\beta_{k}\left(j\right)$ denotes the probability of the partial observation sequence to the right of the position given the genotype $G_{k,j}$ and the map parameters *λ*. The quantities $\alpha_{k}\left(j\right)$ and $\beta_{k}\left(j\right)$ can be obtained using the classical *forward-backward* algorithm (Rabiner 1989; Jiang and Zeng 1997). Their derivation is presented in (Mollinari and Garcia 2019) and briefly described in S1 Extended Material and Methods.

### Offspring haplotype reconstruction

The probability that an offspring individual carries a specific parental homolog at position *k* can be obtained using

$$\text{Pr}\left(H_{k}|O,\lambda \right)=\sum_{j=1}^{400}\text{Pr}\left(H_{k}|G_{k,j},O,\lambda \right)\text{Pr}\left(G_{k,j}|O,\lambda \right)$$ (2)

where, $H_{k}\in \left\{a,b,c,d,e,f,g,h,i,j,k,l\right\}$ is the inherited homolog at locus *k*, $\text{Pr}\left(H_{k}|G_{k,j},O,\lambda \right)=1$ if $H_{k}\in G_{k,j}$, 0 otherwise. We obtained the haplotype probability profile for all 15 homology groups (one curve for each homologs, from *a* to *l*) for all individual in the bi-parental population by computing $\text{Pr}\left(H_{k}|O,\lambda \right)$ at every marker across the genome. For more details, see S1 Extended Material and Methods.

### Heuristic algorithm to detect crossing-over events

Given the probabilistic nature of the haplotype profiles, we proposed the following heuristic algorithm to detect crossing-over events:

1. Regions with haplotype probabilities greater than 0.8 are assumed to be 1.0, otherwise 0.0, forming a binary profile;
2. SNPs within a continuous segment of homolog or gaps flanked by crossing-overs smaller than 10 centimorgans (cM) are removed.
3. If the remaining SNPs represent 20% or more of all SNPs in the analyzed LG, use Equation 1 to re-estimate the 400 genotypes across the whole LG and compute a new homolog probability profile using Equation 2. Otherwise, consider the probability profile inconclusive.
4. The crossing-over points are assessed by checking the points of probability transition across the LG. Homologs involved in the chromosomal exchange can be trivially assessed.
5. Exchange points closer than 0.5 cM are considered inconclusive since the haplotypes involved in the exchange could be erroneously assigned due to the lack of resolution in the mapping population.

We applied this procedure to the 15 LGs of all individuals in the population. We also present an interactive version of the heuristic algorithm at https://gt4sp-genetic-map.shinyapps.io/offspring_haplotype_BT_population/.

### Preferential pairing profiles

Considering that all homologs pair during a hexaploid meiosis, there are 15 possible configurations for a chromosomal segment. Let $\text{Ψ}=\left\{\psi_{i}\right\}$, i=1,…,15 denote a set containing all 15 possible configurations (see S1 Extended Material and Methods). The posterior probability distribution of the pairing configurations at any position in the genome can be computed using

$$\text{Pr}{\left(\psi_{i}|O,\lambda \right)}_{k}=\frac{1}{n}\sum_{l=1}^{n}\sum_{j=1}^{400}\text{Pr}\left(\psi_{i}|G_{k,j},O,\lambda \right)\text{Pr}{\left(G_{k,j}|O,\lambda \right)}_{l}$$ (3)

where *n* is the number of individuals in the population, $\text{Pr}\left(\psi_{i}|G_{k,j},O,\lambda \right)={\left(\frac{m}{2}!\right)}^{-1}$ (m=6 for hexaploids) if $G_{k,j}$ is consistent with $\psi_{i}$, *i.e.*, if genotype $G_{k,j}$ can be originated from the pairing configuration $\psi_{i}$, 0 otherwise (Mollinari and Garcia 2019). We also assessed the preferential pairing for specific homolog pairs $\left(h,h^{\prime }\right)$ using

$$\text{Pr}{\left(h,h^{\prime }|O,\lambda \right)}_{k}=\sum_{i=1}^{15}I_{i}\text{Pr}{\left(\psi_{i}|O,\lambda \right)}_{k},\;h\neq h^{\prime }$$ (4)

where $I_{i}=1$ if $\left(h,h\prime \right)\in \psi_{i}$, 0 otherwise. In both situations, to test whether the observed homolog configurations differ from their expected frequencies under random pairing, we used $\chi^{2}$ test with $P<10^{-4}$ to declare significance. We also used the likelihood associated to recombination fractions of single-dose markers to assess preferential pairing, as suggested by Wu *et al.* (1992). Further details of methods are given in the S1 Extended Material and Methods.

### Data availability

The raw DNA sequences are available on the FTP server of the SweetPotatoBase (ftp://ftp.sweetpotatobase.org/ncsu/). Raw VCF files are available from figshare. File S2 contains SNP locations in *I. trifida* and *I. triloba* reference genomes, available in the Sweetpotato Genomics Resource website (http://sweetpotato.plantbiology.msu.edu). All remaining relevant data are within the manuscript and its Supporting Information files available from figshare. Supplemental material available at figshare: https://doi.org/10.25387/g3.10255844.

## Results

### Genotype calling

**Figure 1:**
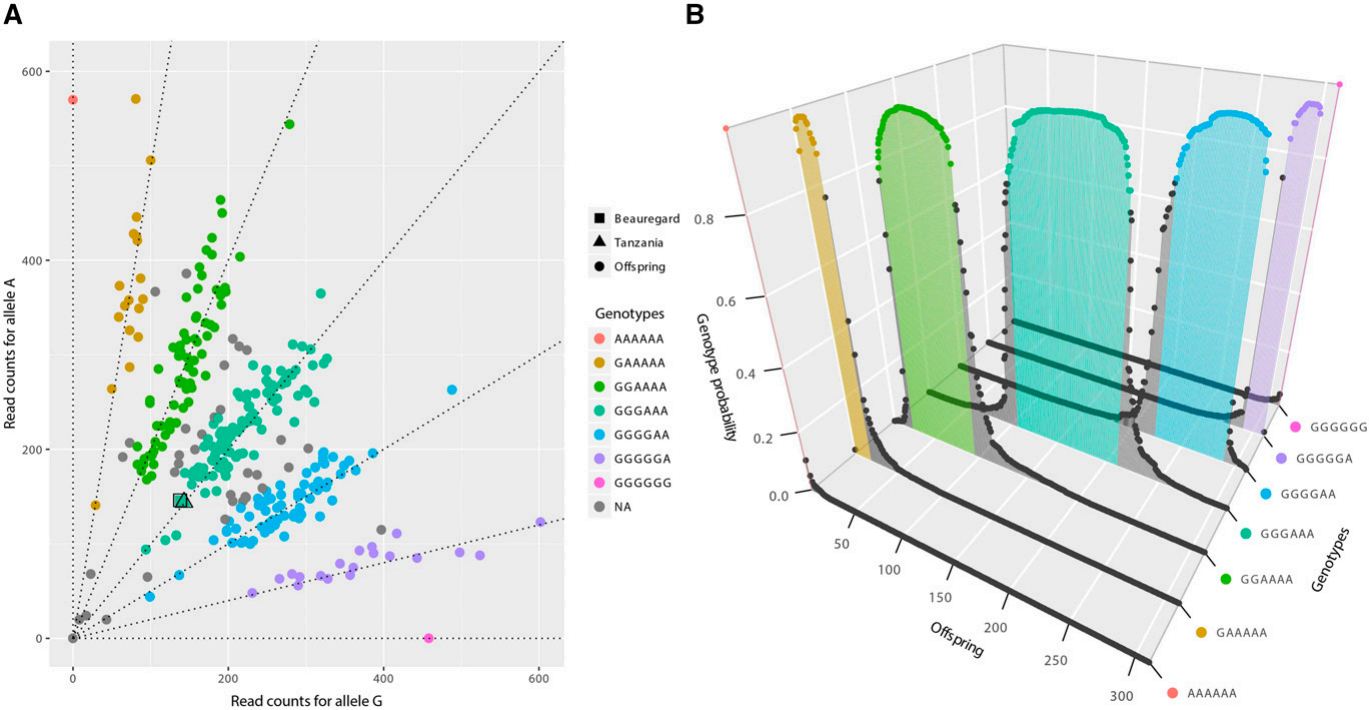
Example of genotype call of SNP Tf_S1_30010438. (A) Scatter plot of the read counts for the two allelic variants A and G. The axes represent the read counts of both allelic variants. Squared and triangle dots represent parents ‘Beauregard’ and ‘Tanzania’ respectively, and regular dots represent the offspring. Dashed lines indicate seven possible dosages in a hexaploid individual. The different colors indicate the dosages assigned to the individuals by SuperMASSA. The low number of individuals observed between genotypic classes (gray dots, with genotype probability smaller than 0.8), outlines a data set with low noise, producing a clear classification. The genotypes of both parents were estimated as three doses of the allelic variant A three doses of G. The genotype calling model also considered the expected Mendelian segregation ratio, which under random chromosome pairing is 1:18:99:164:99:18:1. (B) Inferred probability distribution of genotypes for each individual in the offspring. The colored dots correspond to individuals with the same genotypic classes in panel A. Loci where the highest posterior probability was smaller than 0.8 were assigned as missing data (gray dots).

Next-generation sequencing produced several millions of barcoded reads, resulting in approximately 41 million tags, which were aligned against the genomes of two sweetpotato diploid relatives, *I. trifida* and *I. triloba* (Wu *et al.* 2018), resulting in 1,217,917 and 1,163,397 SNPs, respectively. We used the software SuperMASSA (Serang *et al.* 2012) to call a total of 442,184 SNPs anchored to *I. trifida* genome and 438,808 anchored to *I. triloba* genome. After filtering out low-quality and redundant SNPs (S1 Fig. A), we obtained 38,701 SNPs scored in 311 individuals. SNPs that did not meet the significance threshold ($P<5\times 10^{-4}$) were uniformly distributed across both reference genomes (S2 Fig.). They also presented lower read depth compared with SNPs that passed the threshold, indicating that the distortion observed is rather due to data quality than a biological characteristic of sweetpotato. For all SNPs, we obtained the probability distributions of the dosage calls (exemplified in Figure 1). From the total SNPs, 55.5% were classified as simplex (single-dose markers present in one parent) or double-simplex (single-dose markers present in both parents) and 44.5% were classified as multiplex (S1 Fig. B).

### Initial “de novo” map construction

To build the genetic map, we implemented the R package MAPpoly (https://mmollina.github.io/MAPpoly/). The software comprises routines to perform all steps involved in the map construction of autopolyploid species using a combination of pairwise recombination fraction and HMM-based map estimation. First, we obtained the recombination fractions and associated likelihoods for each possible linkage phase for all SNP pairs (∼749 million pairs). Next, we selected the recombination fractions associated with the most likely linkage phase configuration for each SNP pair and applied the UPGMA hierarchical clustering. We formed 15 distinct clusters representing *I. batatas* homology groups (S3 Fig.). For the 15 groups, 93.4% of the SNPs were co-located on the same chromosomes in both references and LGs (S1 Table). These co-located SNPs were selected to build the initial map. Since each LG had the majority of their SNPs corresponding to a distinct chromosome in both references, LGs were numbered after the diploid references.

To order the SNPs in each LG, we used the MDS algorithm (Preedy and Hackett 2016). The reordered recombination fraction matrix is shown in S4 Fig. A. With the proposed MDS order, the parental allelic variants were phased using the procedure presented by Mollinari and Garcia (2019). The algorithm is based on LOD Scores of pairwise markers as the first source of information to sequentially position the allelic variants in specific homologs. For situations where pairwise analysis had limited power, the algorithm used the likelihood of multiple markers in a Markov chain for the map construction (see Materials and Methods and S1 Extended Material and Methods).

The initial “*de novo*” multilocus map is presented in S4 Fig. B. The length of the LGs ranges from 723.7 cM in LG 8 to 2,037.0 cM in LG 4, with a total map length of 20,201.8 cM and 32,200 SNPs (average inter-locus distance 0.63 cM), with no considerable gaps between SNPs. Although the MDS algorithm yielded adequate global marker orders for all LGs (S4 Fig. C), the resulting map is considerably large. Two main reasons for this inflation are the misplacement of closely linked SNPs and genotyping errors (Cartwright *et al.* 2007; Cheema and Dicks 2009; Bilton *et al.* 2018; Mollinari and Garcia 2019), which will be systematically addressed in the next sections. The alignment of the initial “*de novo*” map against the reference genomes is shown in S5 Fig. Despite several chromosomal rearrangements, we observed high levels of collinearity between both reference genomes and the estimated map. The collinearity extended in blocks with few megabase pairs (Mb), as in LGs 2 and 7, up to the whole chromosome in LGs 5, 9, 10, 11, 12, 14, and 15. In cases where the collinearity extended through the whole chromosome, we observed sites of suppressed recombination (plateaus in S5 Fig.), possibly indicating the location of centromeric regions.

### Reference genome-assisted map improvement and modeling of genotyping errors

To reduce the effects of the local marker misplacement in map inflation, we used *I. trifida* reference genome to propose alternative SNP orders within collinearity blocks and evaluated the likelihood of the resulting maps, keeping the one with the higher likelihood (see Material and Methods and S1 File). We used *I. trifida* as the primary reference genome because the quality of the assembly is superior and more closely related to *I. batatas* when compared to *I. triloba* (Wu *et al.* 2018). After the order improvement, 97.0% of the *I. trifida* SNPs present in the map were locally reordered assuming the *I. trifida* genomic order, (*i.e.*, the genomic order yielded higher likelihoods for the majority of the cases, see Material and Methods and S1 File). From the remaining *I. trifida* SNPs, 1.3% were kept in their initial “*de novo*” order and 1.7% were eliminated since their inclusion caused map inflation higher than 2.00 cM. We then positioned the SNPs private from *I. triloba* reference genome into the resulting map using the constraints imposed by both genomes (see Material and Methods). The reference genome-assisted reordering resulted in a map with 30,723 SNPs spanning 12,937.3 cM with an average inter-locus distance of 0.42 cM, representing a reduction of 1.sixfold when compared to the initial “*de novo*” map (S6 Fig., blue map). To address the effects of genotyping errors, we re-estimated the map using the probability distribution of the genotype callings provided by SuperMASSA (Serang *et al.* 2012) as prior information in the HMM emission function (Mollinari and Garcia 2019), as implemented in MAPpoly (S6 Fig., green map). In this case, the map length was 4,764.1 cM with an average inter-locus distance of 0.16 cM, representing a map reduction of 2.sevenfold when compared to the reference genome-assisted map.

### Probability distribution of multiallelic genotypes and final map estimation

For all individuals in the BT offspring, we obtained the conditional probability distribution of the 400 possible hexaploid genotypes in the whole genome given the estimated genetic map. We used the Markovian process to propagate the information throughout each LG. The genotypic probability distribution at each genome position was assessed by using the information of all markers in the LG in all individuals of the full-sib population (S2 Table and S7 Fig.). Next, we removed 13 individuals with inconsistent genotypic profiles (S8 and S9 Figs.) and, keeping the marker order, we re-estimated the final map considering 298 individuals. A comparison between the initial “*de novo*” and the final maps shows a length reduction of 7.fivefold due to the removal of spurious recombination events through the several steps of map improvement (S6 Fig.).

**Figure 2:**
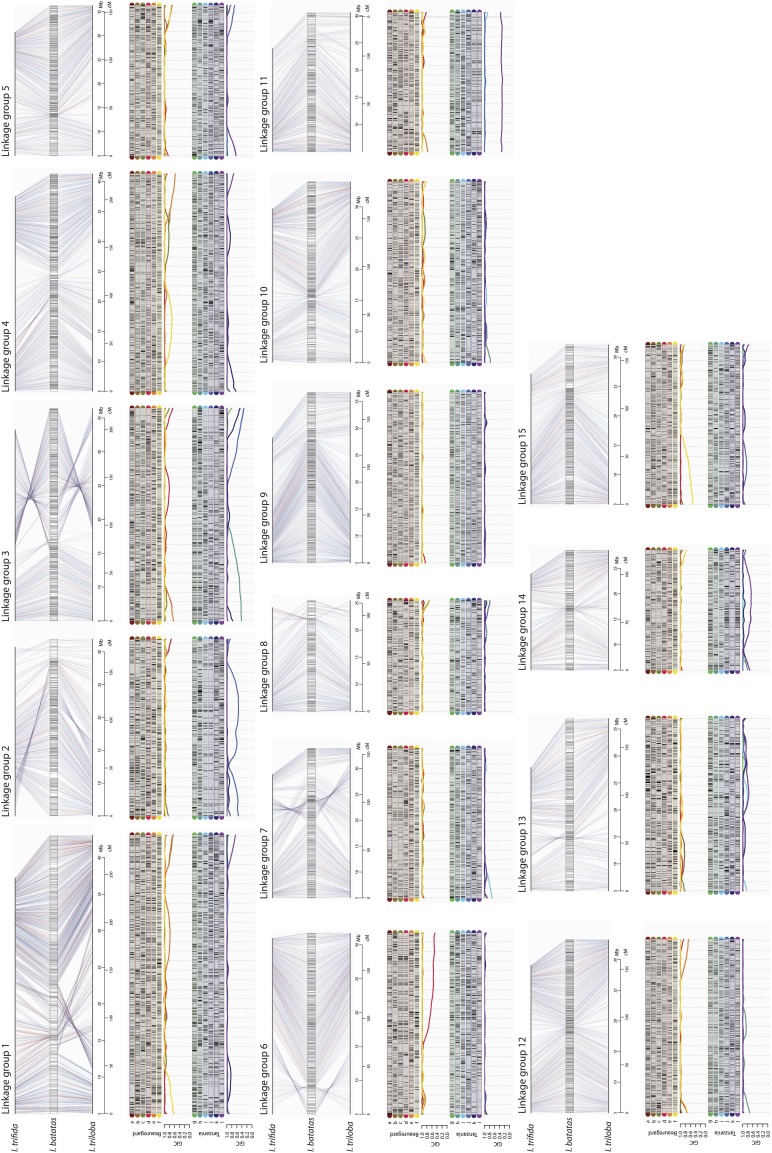
Sweetpotato genetic map. For each of the 15 LGs, we present the *I. batatas* genetic map with its SNPs anchored in both diploid reference genomes. Blue lines connecting the map and reference genomes indicate SNPs shared between *I. trifida* and *I. triloba* reference genomes and red lines indicate private SNPs. Above each map, we present a graphical representation of the parental linkage phase configuration of the homology groups for parents ‘Beauregard’ and ‘Tanzania’. Black and gray rectangles indicate two allelic variants in each marker in all 12 parental homologs (6 × in ‘Beauregard’ and 6 × in ‘Tanzania’). The Genotypic Information Content (GIC), is presented below each homology group.

The final map contains 30,684 SNPs spanning 2,708.4 cM (average inter-locus distance of 0.09 cM), with 60.7% simplex and double-simplex markers, and 39.3% multiplex (Table 1 and Figure 2). All homologs showed allelic variations along the LGs indicating that their inheritance pattern can be assessed in the full-sib population. However, several LG segments presented identical composition for a subset of homologs, as shown by the Genotypic Information Content (GIC) (Bourke *et al.* 2018). In our results, 81.9% of all map positions in ‘Beauregard’ and 77.2% in ‘Tanzania’ had a GIC > 80%, revealing that we can reliably trace back the inheritance of the most homologs from the offspring to the parents (S10 Fig.). A small number of homologs presented an identical allelic composition for certain segments, which is the case, for example, of homologs *i* and *j* for the most of LG 2 and *l* and *k* across the whole LG 11. The complete map can be interactively browsed at https://gt4sp-genetic-map.shinyapps.io/bt_map/. For a selected segment, the browser provides the name of markers, dosages in the parents and the linkage phase configuration of the allelic variants. S2 File shows more map information, including the linkage phase configuration in both parents. S3 and S4 Tables summarize the results of collinearity blocks containing the identical SNP sequences between *I. batatas* genetic map and *I. trifida* and *I. triloba* genomes, respectively. Thirty-nine blocks were aligned to 326.5 Mb of *I. trifida* genome, covering 96.5% of the *I. batatas* map (2,614.8 cM), with an average density of one SNP/14.2 kb; 107 blocks were aligned to 258.8 Mb of *I. triloba* genome, covering 83.1% of the map (2,251.8 cM), with an average density of one SNP/13.4 kb. The averaged genetic to physical map ratios for these regions were of 124.8 kb per cM for *I. trifida* and 114.9 kb per cM for *I. triloba*.

**Table 1:** Summary of sweetpotato genetic map

LG	Length	Number of markers			Total	SNPs/cM
	(cM)	S*^a^*	DS*^b^*	MD*^c^*		
1	290.9	1216	318	1211	2745	9.4
2	184.6	857	197	673	1727	9.4
3	222.1	1085	285	1052	2422	10.9
4	227.1	1374	379	1283	3036	13.4
5	157.1	892	194	815	1901	12.1
6	189.3	970	266	656	1892	10.0
7	156.3	1005	234	612	1851	11.8
8	115.5	712	140	312	1164	10.1
9	178.1	1403	261	715	2379	13.4
10	188.7	1106	234	822	2162	11.5
11	145.6	724	177	729	1630	11.2
12	181.0	1367	246	1048	2661	14.7
13	180.1	762	174	742	1678	9.3
14	125.3	667	96	590	1353	10.8
15	166.6	1019	265	799	2083	12.5
Total	2708.3	15159	3466	12059	30684	11.3

a Simplex markers.

b Double-simplex markers.

c Multiple-dose markers.

### Haplotype reconstruction and multivalent formation

**Figure 3:**
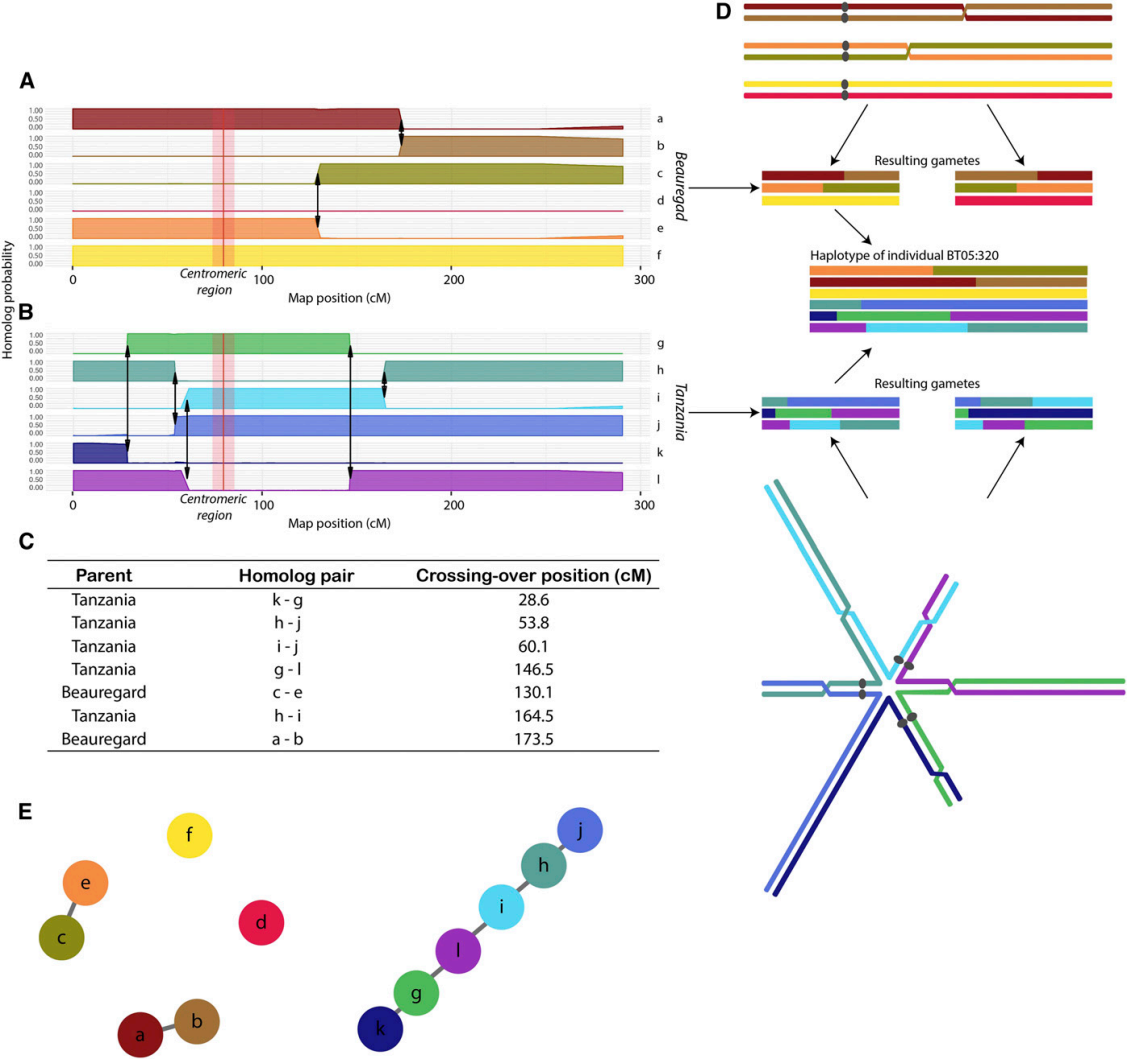
Example of haplotype reconstruction and distribution of meiotic configurations for individual BT05.320, linkage group 1. A) and B) Probability profiles for 12 homologs indicating the segments inherited from parents ‘Beauregard’ and ‘Tanzania’, respectively. The red line indicates the approximated centromeric region obtained using the *I. trifida* reference genome. The arrows indicate recombination points; C) Recombination signature table indicating the homolog pairs involved in each crossing-over and their position in the map; D) Possible meiotic configuration that originated gametes for individual BT05.320 in ‘Beauregard’ and ‘Tanzania’ and resulting gamete. Each chromosome is represented by one chromatid; E) Representation of the meiotic results as a graph: nodes represent the homologs and the edges represent recombination events between them.

To obtain the haplotype composition of all individuals in the full-sib population, we assessed the conditional probability distribution of the genotypes and appropriately combined them to build 12 profiles (one for each homolog) indicating the probability of inheritance of a particular homolog across the whole chromosomes for all individuals in the BT population (see Materials and Methods). The results can be accessed at https://gt4sp-genetic-map.shinyapps.io/offspring_haplotype_BT_population/. By evaluating the recombination points and the homologs involved in the chromosomal exchange, we proposed a heuristic algorithm to obtain chains of homologs linked by recombination events. These chains represent the inference of the meiotic process. The number of parental homologs present in a single homolog of a particular offspring individual indicates the minimum valency of the meiotic configuration involved in its gamete formation (see example in Figure 3).

**Figure 4:**
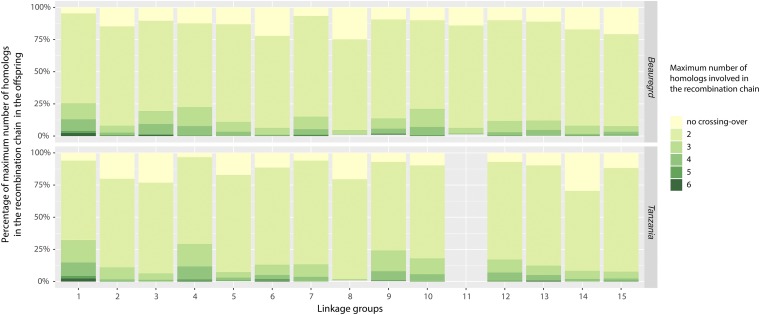
Percentage of maximum number of homologs connected in the same recombination chain during metaphase I in ‘Beauregard’ and ‘Tanzania’ for all 15 LGs. LG 11 for ‘Tanzania’ was mostly inconclusive and is not shown.

Thus, recombination chains with two homologs indicate the formation of at least a bivalent, three homologous, at least a trivalent, and so forth. For each LG, we calculated the percentage of the maximum number of homologs involved in the same recombination chain (Figure 4). Most of the configurations involve recombination of two homologs (73.8% in ‘Beauregard’ and 72.8% in ‘Tanzania’), indicating that there was no evidence of a multivalent formation in the majority of gametes formed. We also observed 12.8% of gametes in ‘Beauregard’ and 15.2% in ‘Tanzania’ with haplotype configurations involving three or four parental homologs in a recombination chain (indicating trivalent or quadrivalent formation), and less than 2% of the meiotic configurations with five or six homologs (indicating pentavalent or hexavalent formation; details per LG in S5 Table). We also detected a significant positive linear correlation ($P<10^{-3}$) between the number of individuals with meiotic configurations originated from multivalent formations and the length of LGs (S11 Fig.).

### Preferential pairing

**Figure 5:**
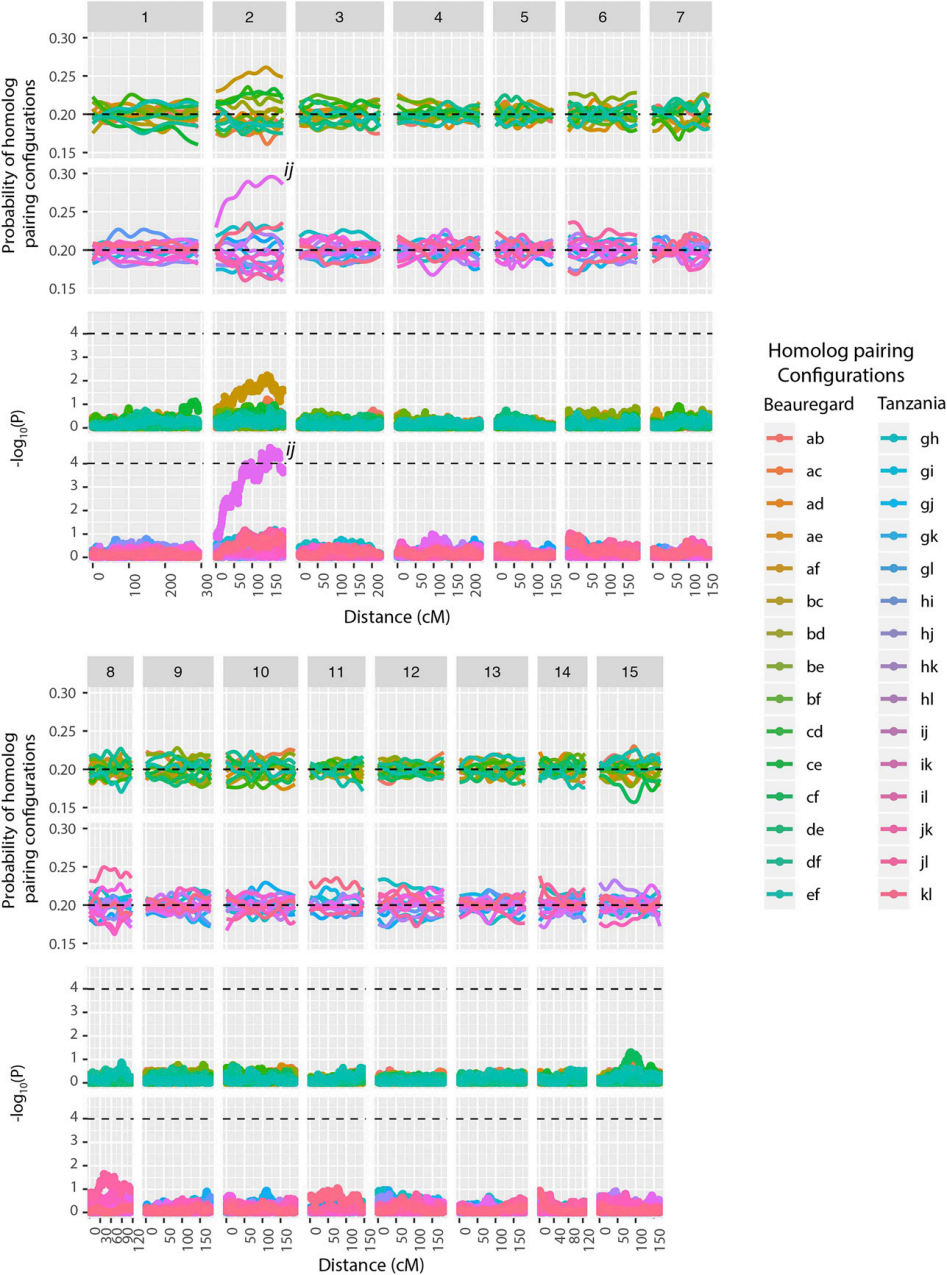
Probability profiles for 15 homolog pairs in parents ‘Beauregard’ and ‘Tanzania’ across 15 LGs. The dashed lines in the probability profiles indicate the pairing probability expected under random pairing ($\frac{3}{15}=0.2$). The lower panels indicate $-{\text{log}}_{10}P$ of a $\chi^{2}$ independence test for all possible homolog pairs. Dashed lines indicate $P<10^{-4}$. Homologs *i* and *j* presented a low, but significant preferential pairing in LG 2.

In a hexaploid organism, there are 15 possible pairing configurations for a chromosome segment during the prophase I of meiosis. To assess the level of preferential pairing among homologs, we calculated the probability profile for each of the 15 possible meiotic pairing configurations (S12 Fig.) and 15 possible homolog pairs (Figure 5) across all LGs for both parents. We did not observe significant preferential pairing across the whole sweetpotato genome, except LG 2 which showed a low but significant preferential pairing between homologs *i* and *j* in parent ’Tanzania’ ($P<10^{-4}$, Figure 5). To further ascertain homolog preferential pairing, we evaluated the simplex marker information, which confirmed our preferential pairing findings using the multilocus framework (S13 Fig.).

## Discussion

We have built the first multilocus integrated genetic map of a hexaploid species, sweetpotato, using our newly developed software MAPpoly. In the map, 90 homologs were densely represented in the 15 homology groups of cultivars ‘Beauregard’ and ‘Tanzania’ exhibiting high collinearity to two closely related diploid sweetpotato genomes, *I. trifida* and *I. triloba*. The high collinearity found by using our ultra-dense map corroborates with the high levels of alignment (> 90%) between the diploid genomes and the parent ‘Tanzania’ reported by Wu *et al.* (2018), suggesting that the diploid genome assemblies could be used as robust references for the hexaploid sweetpotato. We also have constructed the hexaploid haplotypes of all individuals in the offspring, estimating the level of preferential pairing and multivalent formation during the meiotic process at a population level. We used two high-quality reference genomes to improve the quality of our map. However, it is important to notice that in the absence of a reference genome, it is possible to obtain good estimates of the inheritance patterns in the studied population just by using the initial MDS “*de novo*” order and the probability distribution of the genotype calls in the map construction.

Haplotype inference is the ultimate attainment in linkage analysis since it contains the complete information about genome transmission across generations. The challenge of performing such inference both in parents and offspring, would require new approaches to model the multiallelic transmission in a very complex meiotic scenario. Here we accomplished this by propagating the incomplete information of dosage-based SNPs throughout the LG using a Markov chain. As a result of the efficient combination of multiple SNPs, several LGs displayed fully informative parental haplotypes in most of their length (Figure 2 and S10 Fig.). Nevertheless, LG11 had two homologs (*k* and *l*) carrying the same allelic variations across its entire length, which leads us to speculate that these two homologs were formed by nondisjunction of sister chromatids in meiosis II in one of Tanzania’s parent resulting in an unreduced gamete transmitted to the next generation (Burnham 1962). Even though in some cases where not all homologs could be distinguished, we estimated their probability distribution, which can be readily used in further genetic studies, such as quantitative trait loci mapping performed for the BT population (Pereira *et al.* 2019). Moreover, our multipoint method mitigates the effect of a possible limited sample size presented in some studies (Hackett *et al.* 1998; Ripol *et al.* 1999; Doerge and Craig 2000; Luo *et al.* 2001) by using the propagation of the information of multiple markers. In doing so, the estimates of the recombination fractions are obtained considering the structure of the whole homology group, rather pairwise marker combinations. We also investigated how the assembled parental homologs were transmitted to their offspring by assessing the probability distribution of the multiallelic genotypes across the whole genome for all individuals in the mapping population. Based on the inferred probability distributions, we presented a comprehensive probabilistic reconstruction of the haplotypes of all individuals in a full-sib hexaploid population. We found that 15% of the offspring showed the evidence of multivalent formation, *i.e.*, offspring homologs containing more than two parental homologs. This leads to intra-homolog variation, which could not be due to exclusive bivalent pairing.

Multivalent configurations often cause faulty chromosomal segregation leading to aneuploidy (Arana and Nicklas 1992; Hollister 2015). Such a phenomenon could cause unbalanced gametes, and consequently the production of pollen and seeds with low viability, posing a significant hindrance to a stable genomic transmission throughout generations in polyploids (Mwathi *et al.* 2017). Multivalents are usually observed in high numbers in recently formed polyploids, as in the case of the synthetic autopolyploid *Arabidopsis thaliana* (Santos *et al.* 2003). Most of the established autopolyploids, however, show considerably fewer multivalents. In a survey involving 93 autopolyploid species, Ramsey and Schemske (2002) showed that the average frequency of bivalents was 63.7% whereas the average frequency of quadrivalents was 26.8%, which are significantly different from the theoretically expected (1 × two bivalents (II + II) to 2 × one quadrivalent VI) (Sybenga 1975; Jackson and Casey 1982). For hexaploids, the theoretical proportion of bivalent to multivalent configurations is 1 × three bivalents (II + II + II) to 6 × one quadrivalent plus one bivalent (IV + II) to 8 × one hexavalent (VI) (Jackson and Casey 1982). However, in our work, the number of multivalent signatures observed was notably low, whereas the number of bivalents was relatively high (Figure 4). These results corroborate the previous cytological study by Magoon and co-authors (Magoon *et al.* 1970), who found similar levels of multivalent configurations in sweetpotato pachytene cells. Nevertheless, our results provide population-level evidence to the prevalence of bivalent configurations in sweetpotato meiosis.

In a scenario of scarce multivalent formation, the double reduction (DR) phenomenon becomes a rare event. The DR of a given locus is a consequence of a series of cytological events: multivalent formation, crossing-over between the locus and centromere, and migration of the the duplicated segment carrying the locus to the same pole of the cell at anaphases I and II (Mather 1936; Butruille and Boiteux 2000; Stift *et al.* 2008). Thus, multivalent formation is a necessary but not sufficient condition for the occurrence of DR. Consequently, the low frequency of multivalent formation observed in this work indicates that the occurrence of DR is a rare phenomenon in the BT population. Although we did not take into account DR events during the construction of the genetic map, it would have little impact on our results since the algorithm used here was found to be robust under low levels of multivalent formation (Mollinari and Garcia 2019). Nevertheless, although a rare even, DR could generate transgressive genotypes that can be inherited through the next generations.

All sweetpotato genetic maps publish to date (Ukoskit and Thompson 1997; Kriegner *et al.* 2003; Cervantes-Flores *et al.* 2008; Ai-xian *et al.* 2010; Zhao *et al.* 2013; Monden and Tahara 2017; Shirasawa *et al.* 2017) have acknowledged the hexasomic segregation in sweetpotato. However, none of them systematically characterized this phenomenon using the information of multiple markers assembled in complete hexaploid homology groups. Here we used the multilocus map to assess this information generating preferential pairing profiles (Figure 5). We showed that sweetpotato inheritance is vastly autopolyploid-like and random chromosome pairing enables recombination between all homologous across generations. This results are in agreement with studies based on nuclear and chloroplast phylogenies (Roullier *et al.* 2013; Muñoz-Rodríguez *et al.* 2018) which demonstrated the autopolyploid origin of sweetpotato.

A variety of intrachromosomal rearrangements were observed between *I. batatas* map and *I. trifida* and *I. triloba* genomes. Rearrangements mapped to both diploid references, such as the chromosome inversion at the beginning of LG 6 (Figure 2), represent structural changes exclusive to *I. batatas*. While the occurrence of such rearrangements could cause instability to meiotic process at some point of the evolutionary history of a polyploid species (Lenormand *et al.* 2016), given the high level of bivalent signatures and the stable hexasomic segregation observed in our analysis, we concluded that these structural changes became fixed and did not cause major disturbances to the meiotic process in sweetpotato.

More than a linear order of genetic markers positioned in LGs, a genetic map is a statement about the inheritance pattern involved in the transmission of the genome from parents and their offspring. A full characterization of this process can be achieved if the mapping method allows the estimation of haplotypes in both generations. In diploid organisms, a hidden Markov model was proposed by Lander and Green for linkage analysis of multiple markers (Lander and Green 1987). Later on, several studies paved the way for a linkage map construction and haplotype inference in autotetraploid species (Hackett and Broadfoot 2003; Leach *et al.* 2010; Zheng *et al.* 2016). However, for complex polyploids, the map construction was restricted mostly to two-point marker analysis. We present the first integrated multilocus genetic map with fully phased haplotypes for both parents and offspring in a complex polyploid and, accompanied with it, the fully developed statistical methods and computational tool MAPpoly. This opens the door for detailed genetic analysis in complex polyploid species in general.

## References

[bib1] Ai-xian L., Qing-chang L., Qing-mei W., Li-ming Z., Hong Z., 2010 Establishment of Molecular Linkage Maps Using SRAP Markers in Sweet Potato. Zuo Wu Xue Bao 36: 1286–1295.

[bib2] Aitken K. S., Jackson P. A., and McIntyre C. L., 2007 Construction of a genetic linkage map for Saccharum officinarum incorporating both simplex and duplex markers to increase genome coverage. Genome 50: 742–756. 10.1139/G07-056 17893734

[bib3] Arana P., and Nicklas R. B., 1992 Orientation and segregation of a micromanipulated multivalent: Familiar principles, divergent outcomes. Chromosoma 101: 399–412. 10.1007/BF00582834 1618023

[bib4] Austin, D. F., 1988 Exploration, Maintenance and Utilization of Sweet Potato Genetic Resources.No Title. In *Report of the First Sweet Potato Planning Conference*, pp. 27–59, Lima, Peru, International Potato Center.

[bib5] Bilton T. P., Schofield M. R., Black M. A., Chagné D., Wilcox P. L., 2018 Accounting for Errors in Low Coverage High-Throughput Sequencing Data When Constructing Genetic Maps Using Biparental Outcrossed Populations. Genetics 209: 65–76. 10.1534/genetics.117.300627 29487138 PMC5937187

[bib6] Bourke P. M., Gitonga V. W., Voorrips R. E., Visser R. G., Krens F. A., 2018 Multi-environment QTL analysis of plant and flower morphological traits in tetraploid rose. Theor. Appl. Genet. 131: 2055–2069. 10.1007/s00122-018-3132-4 29961102 PMC6154034

[bib7] Burnham C. R., 1962 Discussions in cytogenetics, Burgess Publishing, Mineapolis.

[bib8] Butruille D. V., and Boiteux L. S., 2000 Selection-mutation balance in polysomic tetraploids: impact of double reduction and gametophytic selection on the frequency and subchromosomal localization of deleterious mutations. Proc. Natl. Acad. Sci. USA 97: 6608–6613. 10.1073/pnas.100101097 10823890 PMC18675

[bib9] Cartwright D. A., Troggio M., Velasco R., and Gutin A., 2007 Genetic mapping in the presence of genotyping errors. Genetics 176: 2521–2527. 10.1534/genetics.106.063982 17277374 PMC1950651

[bib10] Cervantes-Flores J. C., Yencho G. C., Kriegner A., Pecota K. V., Faulk M. A., 2008 Development of a genetic linkage map and identification of homologous linkage groups in sweetpotato using multiple-dose AFLP markers. Mol. Breed. 21: 511–532. 10.1007/s11032-007-9150-6

[bib11] Cheema J., and Dicks J., 2009 Computational approaches and software tools for genetic linkage map estimation in plants. Brief. Bioinform. 10: 595–608. 10.1093/bib/bbp045 19933208

[bib12] Comai L., 2005 The advantages and disadvantages of being polyploid. Nat. Rev. Genet. 6: 836–846. 10.1038/nrg1711 16304599

[bib13] Doerge R. W., and Craig B. A., 2000 Model selection for quantitative trait locus analysis in polyploids. Proc. Natl. Acad. Sci. USA 97: 7951–7956. 10.1073/pnas.97.14.7951 10884425 PMC16651

[bib14] FAO, 2017 http://www.fao.org/faostat/en.

[bib15] Fisher R. A., 1941 The theoretical consequences of polyploid inheritance for the mid style form of Lythrum salicaria. Ann. Eugen. 11: 31–38. 10.1111/j.1469-1809.1941.tb02268.x

[bib16] Gallais A., 2003 Quantitative genetics and breeding methods in autopolyploids plants, INRA, Paris.

[bib17] Gemenet, D. C., G. da Silva Pereira, B. De Boeck, J. C. Wood, M. Mollinari, *et al.*, 2019 Quantitative trait loci and differential gene expression analyses reveal the genetic basis for negatively associated *β*-carotene and starch content in hexaploid sweetpotato [*Ipomoea batatas* (l.) lam.]. Theor Appl Genet https://link.springer.com/article/10.1007/s00122-019-03437-7 10.1007/s00122-019-03437-7 PMC6952332 31595335

[bib18] Gustafsson Å., and Gadd I., 1965 Mutations and crop improvement III. *Ipomoea batatas* (L.) Poir. (Convolvulaceae). Hereditas 53: 77–89. 10.1111/j.1601-5223.1965.tb01981.x

[bib19] Hackett C., Bradshaw J. E., Meyer R. C., Mcnicol J. W., Milbourne D., and Waugh R., 1998 Linkage analysis in tetraploid species: a simulation study. Genet. Res. 71: 143–153. 10.1017/S0016672398003188

[bib20] Hackett C. A., and Broadfoot L. B., 2003 Effects of genotyping errors, missing values and segregation distortion in molecular marker data on the construction of linkage maps. Heredity 90: 33–38. 10.1038/sj.hdy.6800173 12522423

[bib21] Hollister J. D., 2015 Polyploidy: Adaptation to the genomic environment. New Phytol. 205: 1034–1039. 10.1111/nph.12939 25729801

[bib22] Jackson R. C., and Casey J., 1982 Cytogenetic Analyses of Autopolyploids: Models and Methods for Triploids to Octoploids. Am. J. Bot. 69: 487–501. 10.1002/j.1537-2197.1982.tb13284.x

[bib23] Jiang C., and Zeng Z.-B., 1997 Mapping quantitative trait loci with dominant and missing markers in various crosses from two inbred lines. Genetica 101: 47–58. 10.1023/A:1018394410659 9465409

[bib24] Kriegner A., Cervantes J. C., Burg K., Mwanga R. O., and Zhang D., 2003 A genetic linkage map of sweetpotato [*Ipomoea batatas*(l.) lam.] based on aflp markers. Mol. Breed. 11: 169–185. 10.1023/A:1022870917230

[bib25] Lander E. S., and Green P., 1987 Construction of multilocus genetic linkage maps in humans. Proc. Natl. Acad. Sci. USA 84: 2363–2367. 10.1073/pnas.84.8.2363 3470801 PMC304651

[bib26] Leach L. J., Wang L., Kearsey M. J., and Luo Z., 2010 Multilocus tetrasomic linkage analysis using hidden markov chain model. Proc. Natl. Acad. Sci. USA 107: 4270–4274. 10.1073/pnas.0908477107 20142473 PMC2840161

[bib27] Lenormand T., Engelstädter J., Johnston S. E., Wijnker E., and Haag C. R., 2016 Evolutionary mysteries in meiosis. Philos. Trans. R. Soc. Lond. B Biol. Sci. 371: 1–14. 10.1098/rstb.2016.0001 PMC5031626 27619705

[bib28] Loebenstein G., 2009 Origin, Distribution and Economic Importance, pp. 9–12 in The Sweetpotato, edited by Loebenstein G., and Thottappilly G. Springer Netherlands, Dordrecht 10.1007/978-1-4020-9475-0_2

[bib29] Luo Z. W., Hackett C. A., Bradshaw J. E., McNicol J. W., and Milbourne D., 2001 Construction of a genetic linkage map in tetraploid species using molecular markers. Genetics 157: 1369–1385. 11238421 10.1093/genetics/157.3.1369 PMC1461571

[bib30] Luo Z. W., Zhang R. M., and Kearsey M. J., 2004 Theoretical basis for genetic linkage analysis in autotetraploid species. Proc. Natl. Acad. Sci. USA 101: 7040–7045. 10.1073/pnas.0304482101 15100415 PMC406462

[bib31] Magoon M. L., Krishnan R., and Vijaya Bai K., 1970 Cytological evidence on the origin of sweet potato. Theor. Appl. Genet. 40: 360–366. 10.1007/BF00285415 24435948

[bib32] Mather K., 1936 Segregation and linkage in autotetraploids. J. Genet. 32: 287–314. 10.1007/BF02982683

[bib33] Mollinari M., and Garcia A. A. F., 2019 Linkage analysis and haplotype phasing in experimental autopolyploid populations with high ploidy level using hidden markov models. G3: Genes, Genomes. Genetics 9: 3297–3314. 10.1534/g3.119.400378 PMC6778803 31405891

[bib34] Mollinari M., and Serang O., 2015 Quantitative SNP Genotyping of Polyploids with MassARRAY and Other Platforms. Methods Mol Biol. 1245: 215–241. 10.1007/978-1-4939-1966-6_17 25373761

[bib35] Monden Y., and Tahara M., 2017 Genetic linkage analysis using DNA markers in sweetpotato. Breed. Sci. 67: 41–51. 10.1270/jsbbs.16142 28465667 PMC5407921

[bib36] Muñoz-Rodríguez P., Carruthers T., Wood J. R., Williams B. R., Weitemier K., 2018 Reconciling Conflicting Phylogenies in the Origin of Sweet Potato and Dispersal to Polynesia. Curr. Biol. 28: 1246–1256.e12. 10.1016/j.cub.2018.03.020 29657119

[bib37] Mwathi M. W., Gupta M., Atri C., Banga S. S., Batley J., 2017 Segregation for fertility and meiotic stability in novel Brassica allohexaploids. Theor. Appl. Genet. 130: 767–776. 10.1007/s00122-016-2850-8 28097399

[bib38] Pereira, G. d. S., D. C. Gemenet, M. Mollinari, B. A. Olukolu, J. C. Wood, *et al.*, 2019 Multiple QTL mapping in autopolyploids: a random-effect model approach with application in a hexaploid sweetpotato full-sib population. bioRxiv p. 10.1101/622951 PMC7337090 32371382

[bib39] Preedy K. F., and Hackett C. A., 2016 A rapid marker ordering approach for high-density genetic linkage maps in experimental autotetraploid populations using multidimensional scaling. Theor. Appl. Genet. 129: 2117–2132. 10.1007/s00122-016-2761-8 27502200

[bib40] Rabiner L., 1989 A tutorial on hidden markov models and selected applications in speech recognition. Proc. IEEE 77: 257–286. 10.1109/5.18626

[bib41] Ramsey J., and Schemske D. W., 2002 Neopolyploidy in Flowering Plants. Annu. Rev. Ecol. Evol. Syst. 33: 589–639. 10.1146/annurev.ecolsys.33.010802.150437

[bib42] Ripol M. I., Churchill G. A., Silva J. A. G. D., and Sorrells M., 1999 Statistical aspects of genetic mapping in autopolyploids. Gene 235: 31–41. 10.1016/S0378-1119(99)00218-8 10415330

[bib43] Roullier C., Duputié A., Wennekes P., Benoit L., Fernández Bringas V. M., 2013 Disentangling the Origins of Cultivated Sweet Potato (*Ipomoea batatas* (L.) Lam.). PLoS One 8: e62707 10.1371/journal.pone.0062707 23723970 PMC3664560

[bib44] Santos J. L., Alfaro D., Armstrong S. J., Franklin F. C. H., and Jones G. H., 2003 Partial Diploidization of Meiosis in Autotetraploid. Genetics 165: 1533–1540. 14668400 10.1093/genetics/165.3.1533 PMC1462840

[bib45] Serang O., Mollinari M., and Garcia A. A., 2012 Efficient exact maximum a posteriori computation for bayesian snp genotyping in polyploids. PLoS One 7: e30906 10.1371/journal.pone.0030906 22363513 PMC3281906

[bib46] Shiotani, I. and T. Kawase, 1987 Synthetic hexaploids derived from wild species related to sweet potato. Japan. J. Breed. 37: 367–376. 10.1270/jsbbs1951.37.367

[bib47] Shirasawa K., Tanaka M., Takahata Y., Ma D., Cao Q., 2017 A high-density SNP genetic map consisting of a complete set of homologous groups in autohexaploid sweetpotato (*Ipomoea batatas*). Sci. Rep. 7: 44207 10.1038/srep44207 28281636 PMC5345071

[bib48] Stift M., Berenos C., Kuperus P., and van Tienderen P. H., 2008 Segregation Models for Disomic, Tetrasomic and Intermediate Inheritance in Tetraploids: A General Procedure Applied to Rorippa (Yellow Cress) Microsatellite Data. Genetics 179: 2113–2123. 10.1534/genetics.107.085027 18689891 PMC2516083

[bib49] Sybenga J., 1975 Meiotic configurations, Springer, Berlin 10.1007/978-3-642-80960-6

[bib50] Ukoskit K., and Thompson P. G., 1997 Autopolyploidy *vs.* allopolyploidy and low-density randomly amplified polymorphic DNA linkage maps of sweetpotato. J. Am. Soc. Hortic. Sci. 122: 822–828. 10.21273/JASHS.122.6.822

[bib51] van Geest G., Bourke P. M., Voorrips R. E., Marasek-Ciolakowska A., Liao Y., 2017 An ultra-dense integrated linkage map for hexaploid chrysanthemum enables multi-allelic qtl analysis. Theor. Appl. Genet. 130: 2527–2541. 10.1007/s00122-017-2974-5 28852802 PMC5668331

[bib52] Wadl P. A., Olukolu B. A., Branham S. E., Jarret R. L., Yencho G. C., 2018 Genetic Diversity and Population Structure of the USDA Sweetpotato (*Ipomoea batatas*) Germplasm Collections Using GBSpoly. Front. Plant Sci. 9: 1166 10.3389/fpls.2018.01166 30186293 PMC6111789

[bib53] Wu K. K., Burnquist W., Sorrells M. E., Tew T. L., Moore P. H., 1992 The detection and estimation of linkage in polyploids using single-dose restriction fragments. Theor. Appl. Genet. 83: 294–300. 10.1007/BF00224274 24202510

[bib54] Wu S., Lau K. H., Cao Q., Hamilton J. P., Sun H., 2018 Genome sequences of two diploid wild relatives of cultivated sweetpotato reveal targets for genetic improvement. Nat. Commun. 9: 4580 10.1038/s41467-018-06983-8 30389915 PMC6214957

[bib55] Yang J., Moeinzadeh M.-H., Kuhl H., Helmuth J., Xiao P., 2017 Haplotype-resolved sweet potato genome traces back its hexaploidization history. Nat. Plants 3: 696–703. 10.1038/s41477-017-0002-z 28827752

[bib56] Zhao N., Yu X., Jie Q., Li H., Li H., 2013 A genetic linkage map based on AFLP and SSR markers and mapping of QTL for dry-matter content in sweetpotato. Mol. Breed. 32: 807–820. 10.1007/s11032-013-9908-y

[bib57] Zheng C., Voorrips R. E., Jansen J., Hackett C. A., Ho J., 2016 Probabilistic multilocus haplotype reconstruction in outcrossing tetraploids. Genetics 203: 119–131. 10.1534/genetics.115.185579 26920758 PMC4858767

[bib58] Zielinski M.-L., and Scheid O. M., 2012 Meiosis in Polyploid Plants, pp. 33–55 in Poliploidy and Genome Evolution, Springer-Verlag, Berlin, Germany.

